# Supplementary material for: Prediction of Ross River virus incidence in Queensland, Australia: building and comparing models
Source: PeerJ. 2022 Nov 8;10:e14213. doi: 10.7717/peerj.14213 (PMC9651042; doi:10.7717/peerj.14213)
Supplement: Text S1 [file peerj-10-14213-s001.docx]

**Supplemental Text S1. Details of data collation and methods**

**Data**

**Table 1. Data source and data collation process**

| **Data^*^** | **Period** | **Temporal / Spatial Unit^*^** | **Data source^*^** | **Data collation steps^**^** | **Final data format** |
| --- | --- | --- | --- | --- | --- |
| **Notification data** |  |  |  |  |  |
| Notified RRV cases | 2001-2020 | Daily / SA3 | QH | - | Continuous |
| Notified RRV cases | 1991-2000 | Total number / SA3 | QH | - | Continuous |
| **Human population** | | | |  |  |
| Human population | 2001-2020 | Yearly / SA3 | ABS | 1;10;11 | Continuous |
| **Climate and environment exposures** | | | |  |  |
| Rainfall (mm) | 2000-2020 | Daily / Grid map | BOM | 1;3;4;5;9;11;12 | Continuous |
| Maximum air temperature (°C) | 2000-2020 | Daily / Grid map | BOM | 1;3;4;5;9;7;11;12 | Continuous |
| Range of air temperature (°C) | 2000-2020 | Daily / Grid map | BOM | 1;3;4;5;9;7;11;12 | Continuous |
| Relative humidity at the time of maximum temperature (%) | 2000-2020 | Daily / Grid map | BOM | 1;3;4;5;9;11;12 | Continuous |
| Relative humidity at the time of minimum temperature (%) | 2000-2020 | Daily / Grid map | BOM | 1;3;4;5;9;11;12 | Continuous |
| Pan Evaporation (mm) | 2000-2020 | Daily / Grid map | BOM | 1;3;4;5;9;11;12 | Continuous |
| Evapotranspiration (mm) | 2000-2020 | Daily / Grid map | BOM | 1;3;4;5;9;11;12 | Continuous |
| Vapour pressure at 9 AM (hPa) | 2000-2020 | Daily / Grid map | BOM | 3;4;5;9;11 | Continuous |
| Vapour pressure at 3 PM (hPa) | 2000-2020 | Daily / Grid map | BOM | 3;4;5;9;11 | Continuous |
| El Niño events | 2000-2020 | Monthly / QLD | BOM | 2 | Binary |
| La Niña events | 2000-2020 | Monthly / QLD | BOM | 2 | Binary |
| Southern Oscillation Index | 2000-2020 | Monthly / QLD | BOM | 11 | Continuous |
| **Geographical exposures** | | | |  |  |
| Normalized Difference Vegetation Index | 2000-2020 | Monthly / Grid map | BOM | 11 | Continuous |
| Flooding | 2000-2020 | Daily / SA3 | BOM | 2 | Binary |
| Bushfire | 2000-2020 | Daily / SA3 | BOM | 2 | Binary |
| Land use (%) | 2019 | Constant / Feature map | Q Spatial | 6;16 | Continuous |
| Lakes (%) | 2019 | Constant / Feature map | Q Spatial | 6;16 | Continuous |
| Reservoirs (%) | 2019 | Constant / Feature map | Q Spatial | 6;16 | Continuous |
| Wetlands (%) | 2019 | Constant / Feature map | Q Spatial | 6;13;14;15;16 | Continuous |
| Elevation (m) | 2008 | Constant / Point map | GA | 3;4;8;12 | Continuous |
| Coastal areas | - | Constant / SA3 | ABS | - | Binary |
| **Socio-economic exposures** | | | |  |  |
| The Socio-Economic Index for Areas | 2011, 2016 | 2011, 2016 / SA3 | ABS | 11 | Continuous |
| Accessibility / Remoteness Index of Australia | 2011, 2016 | 2011, 2016 / SA3 | Hugo Centre | 11 | Categorical |

^*^ RRV = Ross River virus; SA3 = Statistical Area Level 3; QH = Queensland Department of Health; ABS = Australian Bureau of Statistics; BOM = Australian Bureau of Meteorology; Q Spatial = Queensland Spatial Catalogue; GA = The Geoscience Australia website.

^**^ 1. Extract data from NetCDF files; 2. Extract data from Significant Weather Summaries of Queensland; 3. Convert grid maps to point maps; 4. Spatial join the point data with Queensland map at SA3 areas; 5. Extract data in each area; 6. Spatial splitting the data according to Queensland map with SA3 area boundaries; 7. Combine the data sets of maximum and minimum exposure value, calculate the daily range of the exposure; 8. Calculate average value for each area; 9. Calculate daily average value for each area; 10. Linear interpolation / extrapolation; 11. Calculate weekly average data; 12. Spatial interpolation using average of adjacent grid points (Nearest Neighbours) for the areas having missing values; 13. Remove the marine wetlands; 14. Remove the wrongly classified wetlands; 15. The areas of some wetlands (> 100 hectares) were adjusted based on their shape area in ArcGIS by the median ratio of wetland area and shape area before splitting; 16. Calculate the percentage of land areas for each area.

**Model building**

**Generalised Linear Models**

The generalised linear models applied in this study included Standard Poisson generalised linear model (Poisson) and Standard Negative Binomial generalised linear model (NB).

Generalised linear models are widely applied in predicting Ross River virus (RRV) notifications, incidence rates and outbreaks [1]. Linear models are simple and straightforward in explaining the relationship between exposures and RRV infection outcome variables. We developed a Poisson model based on the assumption that the log transformed RRV incidence follows a Poisson distribution and can be predicted by a linear combination of independent exposures. However, RRV data do not always follow the assumption of the Poisson distribution that the variance equals the mean. A NB model is more tolerant to variation from this assumption. So, NB models can fit the over-dispersed count data with variance exceeding the mean.

A generalised linear model consists of a random component, a linear predictor and a smooth and invertible linearising link function [2],

$g(E(Y_{i}))=\alpha+\beta_{1}X_{i1}+\beta_{2}X_{i2}+\cdots+\beta_{K}X_{iK}$ (1)

where is the link function for both Poisson and NB models, $E(Y_{i})$ is the expectation of the response variable for the *i*th observation, is the variable and *K* is the number of variable, $\alpha$ is the intercept, $\beta_{K}$ is the coefficient of the *K*th variable.

**Zero-Inflated models**

Models including Zero-Inflated Poisson model with constant in zero-part (ZIP), Zero-Inflated Poisson model with exposure as a regressor in zero-part (ZIPe), Zero-Inflated Negative Binomial model with constant in zero-part (ZINB), and Zero-Inflated Negative Binomial model with exposure as a regressor in zero-part (ZINBe) were used in this study.

A zero-inflated model consists of a logit part which allows for excess zeros in the data, and a linear regression part that has a given distribution (such as Poisson or Negative Binomial). Given the weekly data used in this study included numerous zeros, the ZIP model and ZINB models are appropriate for analysing data with excess zeros [3, 4].

The Zero-inflated model [3, 4] is:

(2)

The weight determines the proportion of excess zeros for the *i*th observation. The $g(y)$ in Zero-inflated Poisson model is:

$g(y)=\frac{e^{-\lambda}\lambda^{y}}{y!}$ (3)

where $\lambda_{i}=e^{x_{i}\beta}$ and $x_{i}$ is the regression variables in the *i*th observation and $\beta$is the vector of regression coefficients. The $g(y)$ in Zero-inflated Negative Binomial model [5] is:

$g(y)=\frac{\Gamma(y+1/\alpha)}{\Gamma(y+1)\Gamma(1/\alpha)}\left(\frac{1}{1+\alpha\lambda}\right)^{1/\alpha}\left(\frac{\alpha\lambda}{1+\alpha\lambda}\right)^{y}$ (4)

In zero-inflated model, if the zero component of the model includes no exposures, the model assumes that all zeros in the data have the same probability of being excess zeros. Otherwise, if an exposure is included in the zero component, the exposure is assumed to distinguish whether RRV cases occur or not, thus it is used as a regressor to identify excess zeros from all zeros. Considering that weather exposures predict RRV incidence well and lags in three months are epidemiological reasonable, the lagged variables of weather exposures up to 13 weeks were generated using the total data in Queensland across all years. The areas under curve (AUC) of these variables for predicting the presence of RRV were calculated. The variable with highest AUC was applied in the zero-part of the zero-inflated models. Linear models with or without exposure in the zero-part were built.

**Generalised Additive Models**

The Poisson Generalised Additive Model (PGAM), Negative Binomial Generalised Additive Model (NBGAM), Zero-Inflated Poisson Generalised Additive Model (ZIPGAM), and Zero-Inflated Negative Binomial Generalised Additive Model (ZINBGAM) were implemented in this study.

The complicated relationship between lagged exposures and RRV incidence indicate possible existence of non-linear effects. The generalised additive model can include linear and non-linear exposure terms [6].

The generalised additive model is given by:

$g(E(Y))=\alpha+f_{1}(X_{1})+f_{2}(X_{2})+\cdot\cdot\cdot+f_{m}(X_{m})$ (5)

where $g(E(Y))$ is the link function of a family of distributions, including normal, binomial, and negative binomial, $X_{i}$ can be a univariate or multivariate covariate, $f_{i}$ can be a simple parametric function, or a smooth non-parametric or semi-parametric function such as a smoothing spline, natural cubic spline or local regression.

Here we used natural cubic splines to fit the 3^rd^ degree polynomials to data with maximum of three knots. The total data in Queensland across all years were used to calculate the exposures and their lags in one year. Then these variables and natural cubic spline with up to three knots of these variables were used to fit the model of RRV incidence separately. The trends of actual RRV trends and predicted RRV trends with the variables were displayed. The performance of the models was calculated to evaluate the model fit in training data where the model is generated, and in validation data where the model is tested with new data. Whether to use natural cubic spline for each variable and the best knot were decided according to a balance between better model fit and lower complexity (with fewer unknown parameters or predictors).

Non-linear terms were added to both generalised linear models and ZI models to evaluate the effect of non-linearity. In generalised additive models with zero-inflation, no exposures were included in the zero-part because a variable which can well predict the presence of RRV in this kind of model might lead to an identifiability problem [7].

**Table 2. A summary of the models used in this study.**

| **Model** | **Abbreviation** | **Model assumption** |
| --- | --- | --- |
| Standard Poisson generalised linear model | Poisson | The data follows Poisson distribution. |
| Standard Negative Binomial generalised linear model | NB | The data follows negative binomial distribution. |
| Zero-Inflated Poisson model with constant in zero-part | ZIP | The data follows Poisson distribution, and all zeros in the data have the same probability of being excess zeros. |
| Zero-Inflated Poisson model with an exposure as a regressor in zero-part | ZIPe | The data follows Poisson distribution, and the probability of being excess zeros from all zeros is based on a regressor. |
| Zero-Inflated Negative Binomial model with constant in zero-part | ZINB | The data follows negative binomial distribution, and all zeros in the data have the same probability of being excess zeros. |
| Zero-Inflated Negative Binomial model with an exposure as a regressor in zero-part | ZINBe | The data follows negative binomial distribution, and the probability of being excess zeros from all zeros is based on a regressor. |
| Poisson Generalised Additive Model | PGAM | The data follows Poisson distribution, and some variables have non-linear correlations with the outcome. |
| Negative Binomial Generalised Additive Model | NBGAM | The data follows negative binomial distribution, and some variables have non-linear correlations with the outcome. |
| Zero-Inflated Poisson Generalised Additive Model | ZIPGAM | The data follows Poisson distribution, all zeros in the data have the same probability of being excess zeros, and some variables have non-linear correlations with the outcome. |
| Zero-Inflated Negative Binomial Generalised Additive Model | ZINBGAM | The data follows negative binomial distribution, all zeros in the data have the same probability of being excess zeros, and some variables have non-linear correlations with the outcome. |

**References:**

1. Qian W, Viennet E, Glass K, Harley D. Epidemiological models for predicting Ross River virus in Australia: A systematic review. PLoS neglected tropical diseases. 2020;14(9):e0008621.
2. Fox J. Applied regression analysis and generalized linear models: Sage Publications; 2015.
3. Lambert D. Zero-inflated Poisson regression, with an application to defects in manufacturing. Technometrics. 1992;34(1):1-14.
4. Lim HK, Li WK, Philip L. Zero-inflated Poisson regression mixture model. Computational Statistics & Data Analysis. 2014;71:151-8.
5. Yau KK, Wang K, Lee AH. Zero‐inflated negative binomial mixed regression modeling of over‐dispersed count data with extra zeros. Biometrical Journal: Journal of Mathematical Methods in Biosciences. 2003;45(4):437-52.
6. Ma W, Sun X, Song Y, Tao F, Feng W, He Y, et al. Applied mixed generalized additive model to assess the effect of temperature on the incidence of bacillary dysentery and its forecast. 2013;8(4):e62122.
7. Wood S. mgcv: Mixed GAM Computation Vehicle with Automatic Smoothness Estimation. 2021. [cited 03-December-2021] Available from: <https://mran.microsoft.com/web/packages/mgcv/index.html>.
