# Supplementary material for: Prediction of Ross River virus incidence in Queensland, Australia: building and comparing models
Source: PeerJ. 2022 Nov 8;10:e14213. doi: 10.7717/peerj.14213 (PMC9651042; doi:10.7717/peerj.14213)
Supplement: Table S1 — * (a) using the same model to select variable sets and to do prediction; (b) using a specific model (NB) to select variable sets and then applied the variable set in all models. ** Poisson: standard Poisson generalised linear model, NB: standard negative binomial generalised linear model, ZIP: zero-inflated Poisson model with constant in zero part, and ZIPe: zero-inflated Poisson model with an exposure as a regressor in zero part. [file peerj-10-14213-s002.docx]

**Supplemental Table S1.** **Model fit of using a) method and b) method in building** **Poisson models, NB models, ZIP models, and ZIPe models in different regions of Queensland.**

| **Region** | **Model fit criteria** | **NB^**^** | **using a) method for selecting variables^*^** | | | **using b) method (NB) for selecting variables^*^** | | |
| --- | --- | --- | --- | --- | --- | --- | --- | --- |
|  |  |  | **Poisson^**^** | **ZIPe^**^** | **ZIP^**^** | **Poisson^**^** | **ZIPe^**^** | **ZIP^**^** |
| All | AIC | 82422 | 88458 | 85827 | Not studied^^^ | 88519 | 85785 | 85819 |
| All | BIC | 82560 | 88590 | 85942 | Not studied^^^ | 88648 | 85932 | 85957 |
| All | HQIC | 82426 | 88463 | 85832 | Not studied^^^ | 88525 | 85792 | 85825 |
| Dry | AIC | 8052 | 8810 | 8595 | 8837 | 8589 | 8328 | 8327 |
| Dry | BIC | 8118 | 8865 | 8655 | 8903 | 8648 | 8400 | 8394 |
| Dry | HQIC | 8051 | 8811 | 8596 | 8821 | 8590 | 8329 | 8329 |
| Hot | AIC | 20022 | 21449 | 20949 | 21362 | 21365 | 20923 | 20923 |
| Hot | BIC | 20120 | 21538 | 21036 | 21477 | 21456 | 21028 | 21021 |
| Hot | HQIC | 20023 | 21452 | 20951 | 21334 | 21368 | 20926 | 20926 |
| Warm | AIC | 52789 | 55838 | 54841 | 55685 | 55890 | 54033 | 54050 |
| Warm | BIC | 52928 | 55997 | 54971 | 55816 | 56020 | 54180 | 54189 |
| Warm | HQIC | 52793 | 55845 | 54846 | 55659 | 55895 | 54039 | 54056 |

^*^a) using the same model to select variable sets and to do prediction; b) using a specific model (NB) to select variable sets and then applied the variable set in all models. ^**^Poisson: standard Poisson generalised linear model, NB: standard negative binomial generalised linear model, ZIP: zero-inflated Poisson model with constant in zero part, and ZIPe: zero-inflated Poisson model with an exposure as a regressor in zero part. ^^^We started the formal analysis after preliminary study had provided enough information to support the use of NB models for variable selection in this study.
