# Supplementary material for: Prediction of Ross River virus incidence in Queensland, Australia: building and comparing models
Source: PeerJ. 2022 Nov 8;10:e14213. doi: 10.7717/peerj.14213 (PMC9651042; doi:10.7717/peerj.14213)
Supplement: Table S2 [file peerj-10-14213-s003.docx]

**Supplemental Table S2. The relative residual measures of all models in validation sets.**

| **Region** | **Relative residual^*^** | **Poisson^**^** | **NB^**^** | **ZIPe^**^** | **ZINBe^**^** | **ZIP^**^** | **ZINB^**^** | **PGAM^**^** | **NBGAM^**^** | **ZIPGAM^**^** | **ZINBGAM^**^** |
| --- | --- | --- | --- | --- | --- | --- | --- | --- | --- | --- | --- |
| All | MAE | 0.743 | 0.744 | 0.729 | 0.744 | 0.729 | 0.744 | 0.795 | 0.788 | 1.010 | 0.806 |
| Dry | MAE | 0.878 | 0.884 | 0.832 | 0.882 | 0.830 | 0.884 | 0.904 | 0.931 | 1.141 | 0.963 |
| Hot | MAE | 0.972 | 0.973 | 0.939 | 0.972 | 0.940 | 0.973 | 0.998 | 0.990 | 1.099 | 0.990 |
| Warm | MAE | 0.681 | 0.677 | 0.671 | 0.676 | 0.672 | 0.677 | 0.735 | 0.714 | 0.976 | 0.722 |
| All | MSE | 1.656 | 1.742 | 1.555 | 1.742 | 1.554 | 1.743 | 1.886 | 1.853 | 2.786 | 1.978 |
| Dry | MSE | 1.769 | 1.785 | 1.492 | 1.780 | 1.484 | 1.785 | 1.854 | 1.953 | 2.871 | 2.141 |
| Hot | MSE | 2.722 | 2.718 | 2.457 | 2.773 | 2.458 | 2.718 | 2.779 | 2.689 | 3.209 | 2.690 |
| Warm | MSE | 1.428 | 1.412 | 1.388 | 1.407 | 1.393 | 1.412 | 1.610 | 1.510 | 2.472 | 1.537 |
| All | RMSE | 1.272 | 1.310 | 1.230 | 1.310 | 1.230 | 1.310 | 1.352 | 1.348 | 1.619 | 1.388 |
| Dry | RMSE | 1.296 | 1.302 | 1.209 | 1.300 | 1.205 | 1.301 | 1.331 | 1.368 | 1.636 | 1.432 |
| Hot | RMSE | 1.645 | 1.642 | 1.566 | 1.660 | 1.566 | 1.642 | 1.660 | 1.632 | 1.781 | 1.632 |
| Warm | RMSE | 1.163 | 1.160 | 1.144 | 1.157 | 1.146 | 1.160 | 1.235 | 1.202 | 1.525 | 1.213 |

^*^MAE = Mean Absolute Error; MSE = Mean Square Error; RMSE = Root Mean Square Error.

^**^ Poisson =Standard Poisson generalised linear model; NB = Standard Negative Binomial generalised linear model; ZIP = Zero-Inflated Poisson model with constant in zero part; ZIPe = Zero-Inflated Poisson model with exposure as regressor in zero part; ZINB = Zero-Inflated Negative Binomial model with constant in zero part; ZINBe = Zero-Inflated Negative Binomial model with exposure as regressor in zero part; PGAM = Poisson Generalized Additive Model; NBGAM = Negative Binomial Generalized Additive Model; ZIPGAM = Zero-Inflated Poisson Generalized Additive Model; ZINBGAM = Zero-Inflated Negative Binomial Generalized Additive Model.
