# Supplementary figures and images for: Prediction of Ross River virus incidence in Queensland, Australia: building and comparing models
Source: PeerJ. 2022 Nov 8;10:e14213. doi: 10.7717/peerj.14213 (PMC9651042; doi:10.7717/peerj.14213)

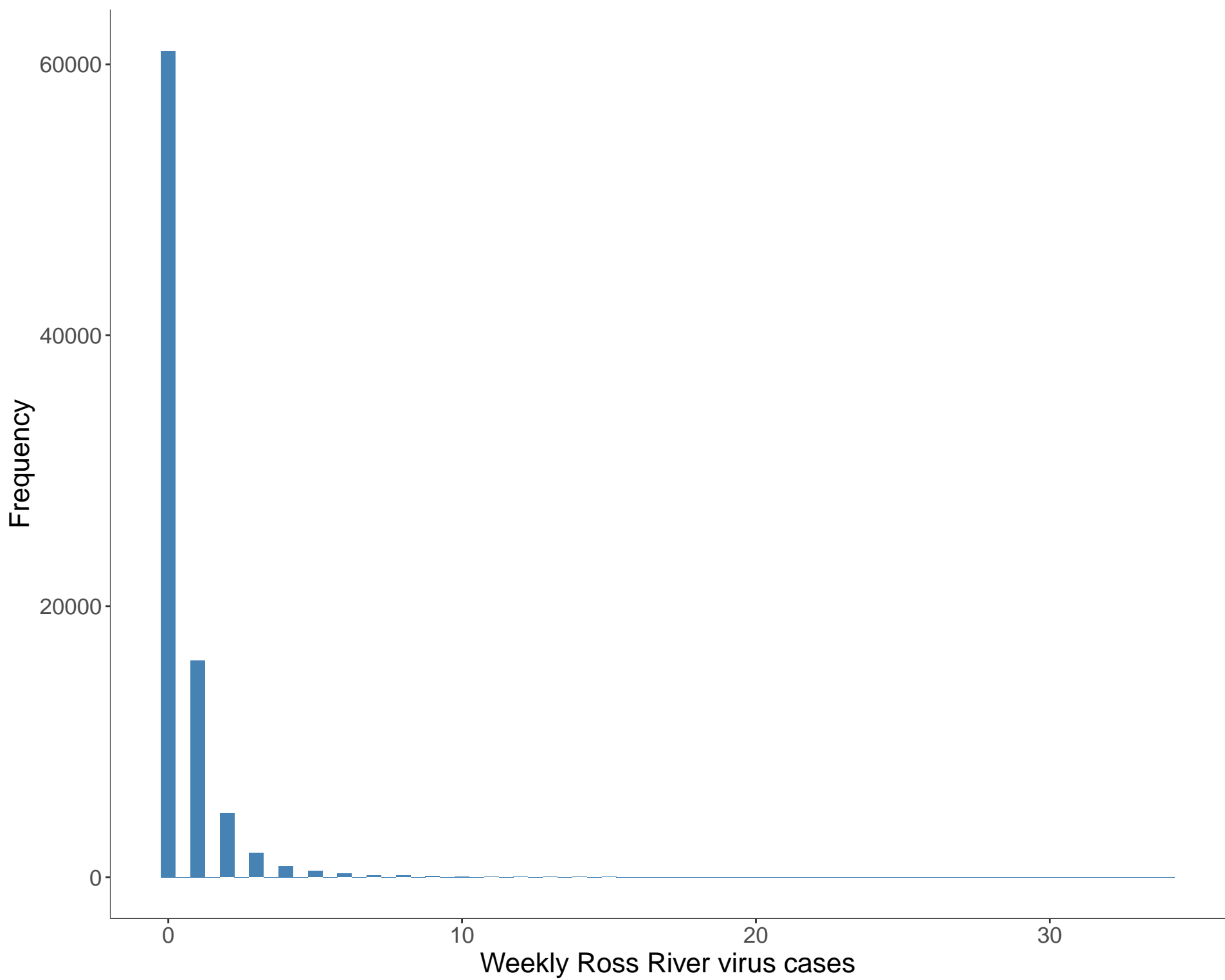

Supplement: Figure S1 [file peerj-10-14213-s004.pdf]

Linear models ZI models Non-linear models

All

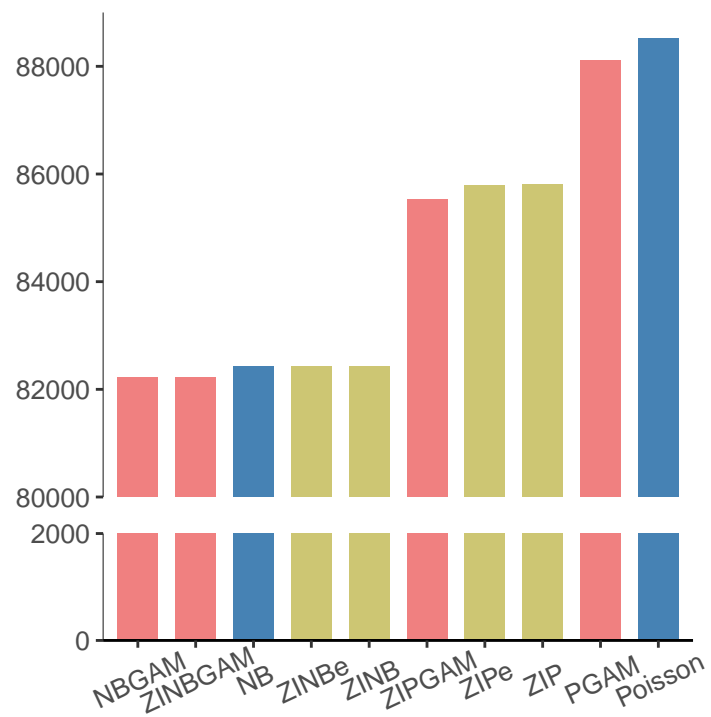

Warm

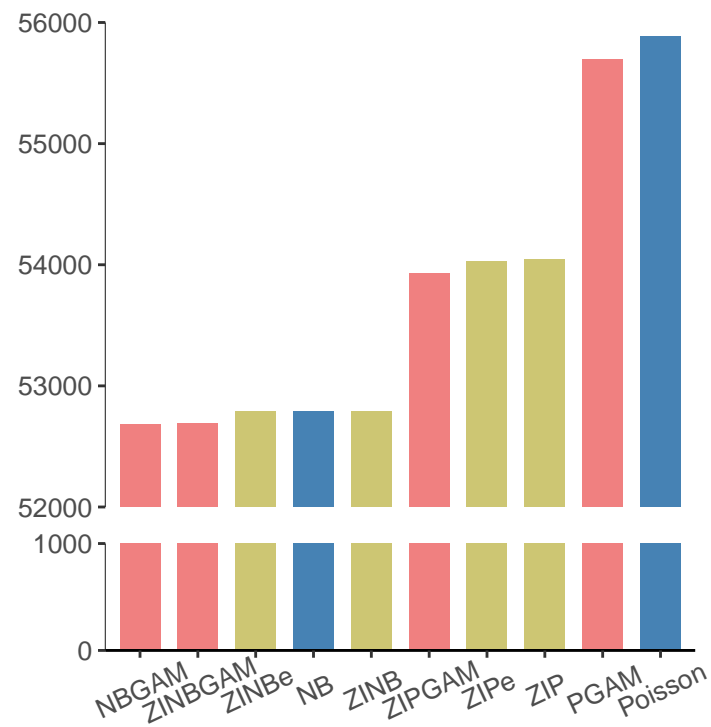

Dry

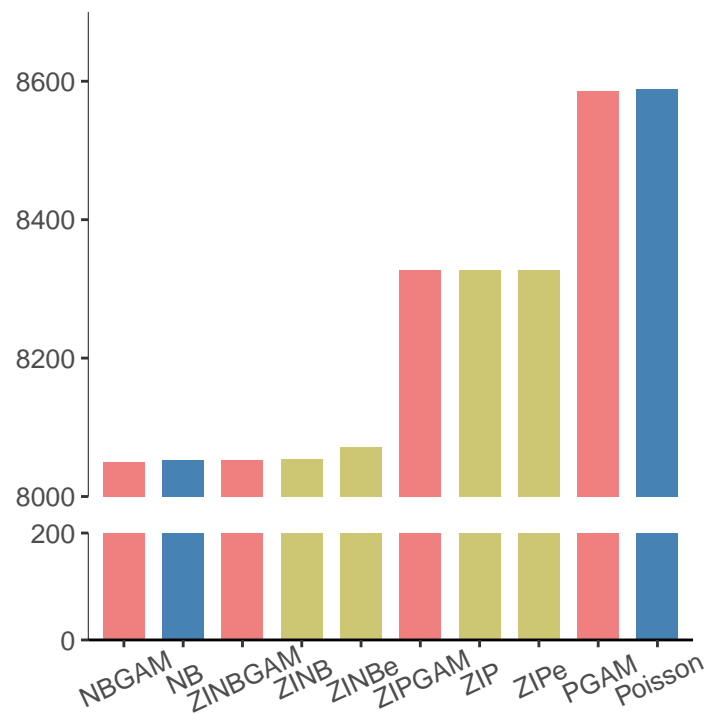

Hot

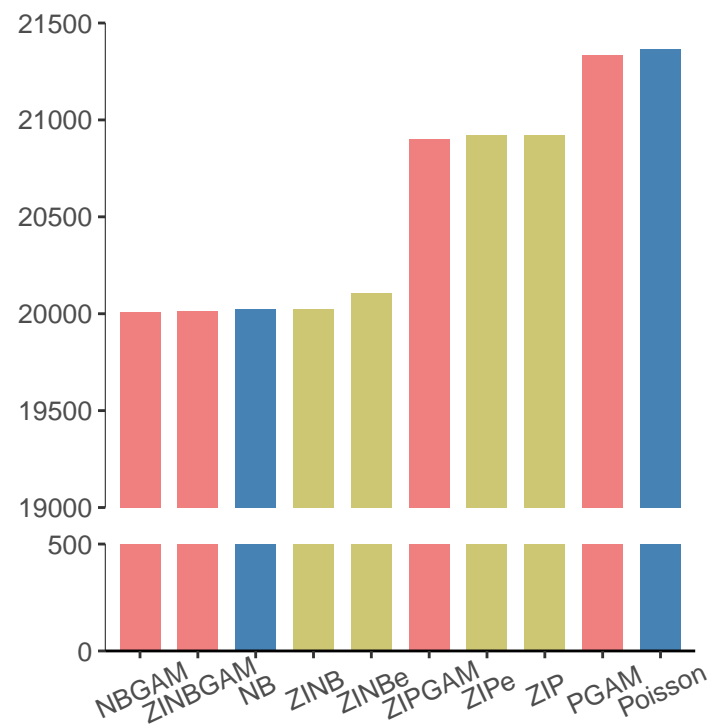

Method

Supplement: Figure S2 [file peerj-10-14213-s005.pdf]

Linear models ZI models Non-linear models

All

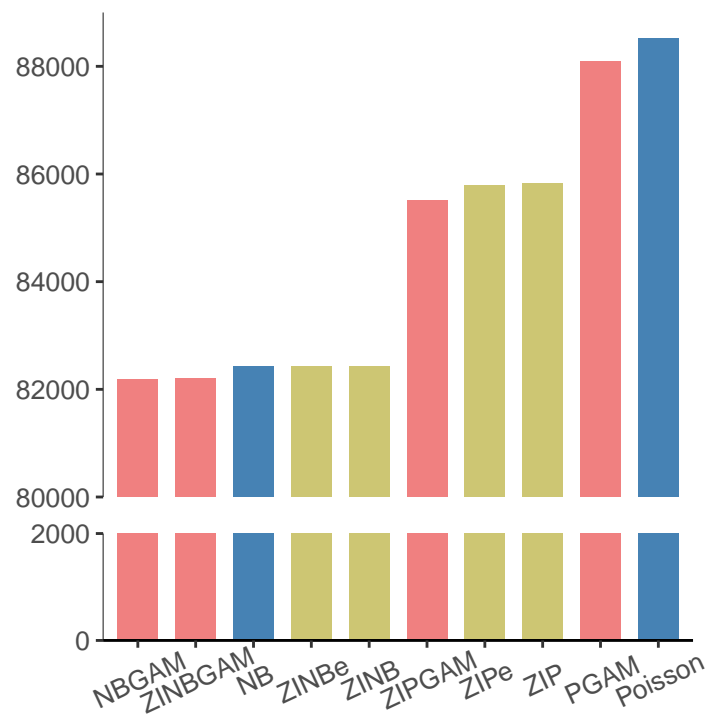

Warm

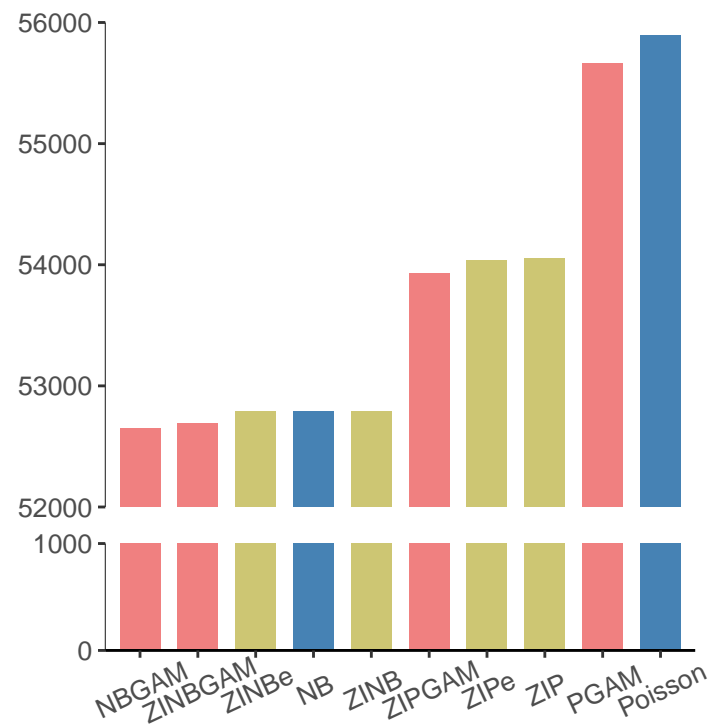

Dry

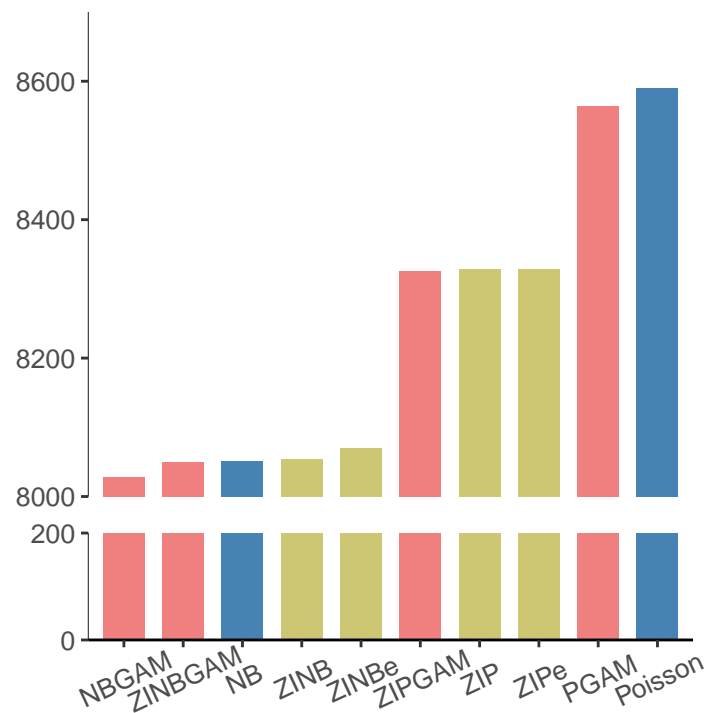

Hot

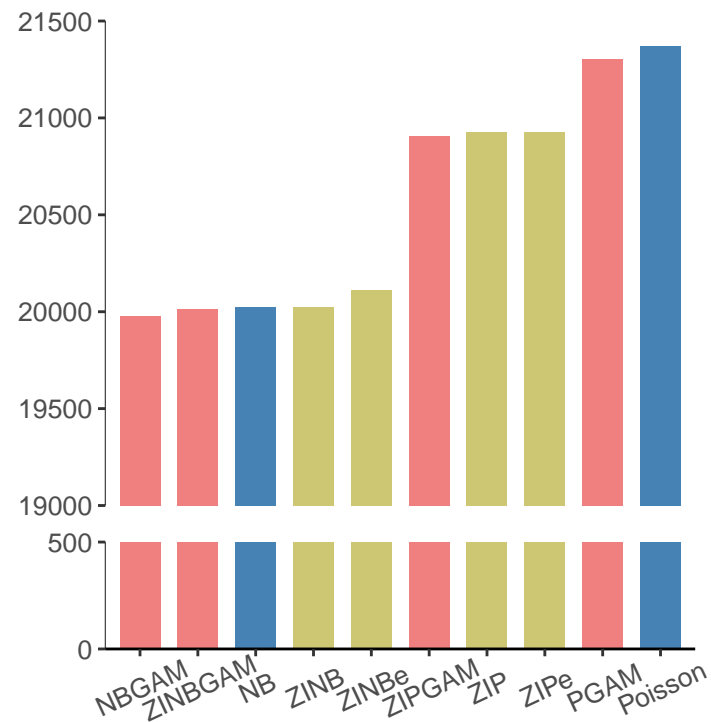

Method

HQIC

Supplement: Figure S3 [file peerj-10-14213-s006.pdf]

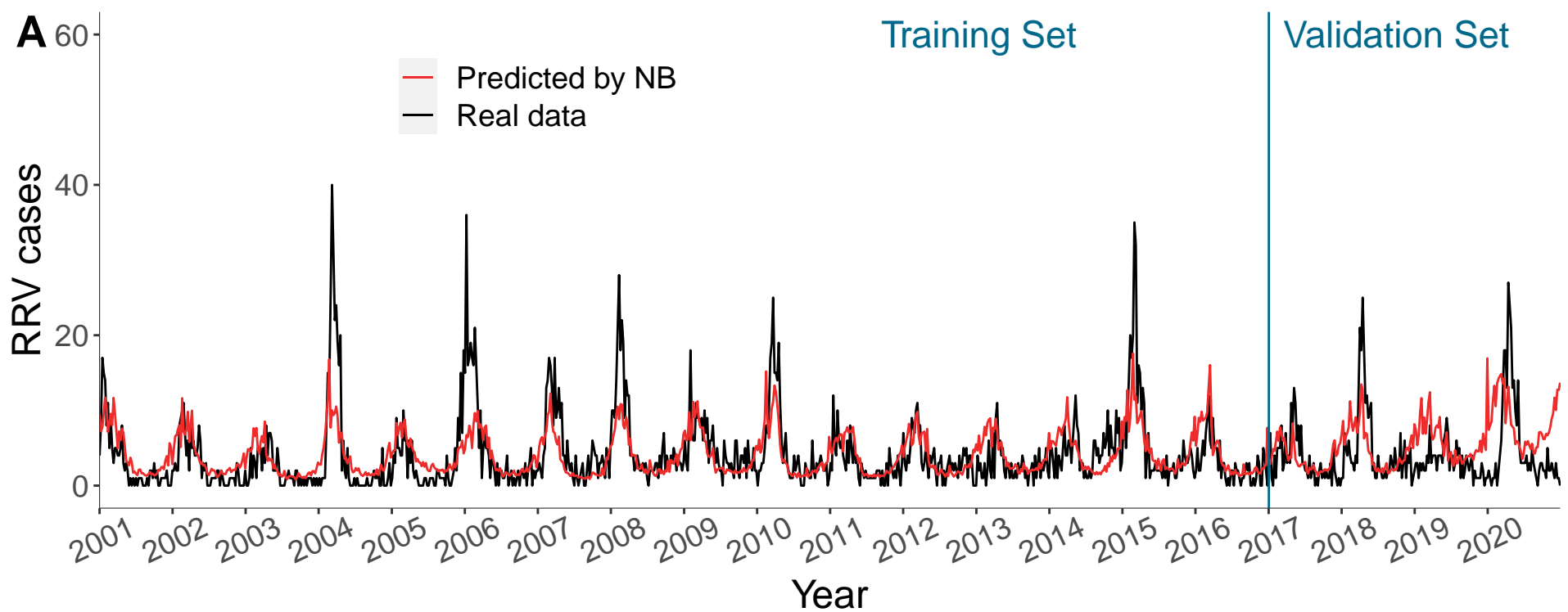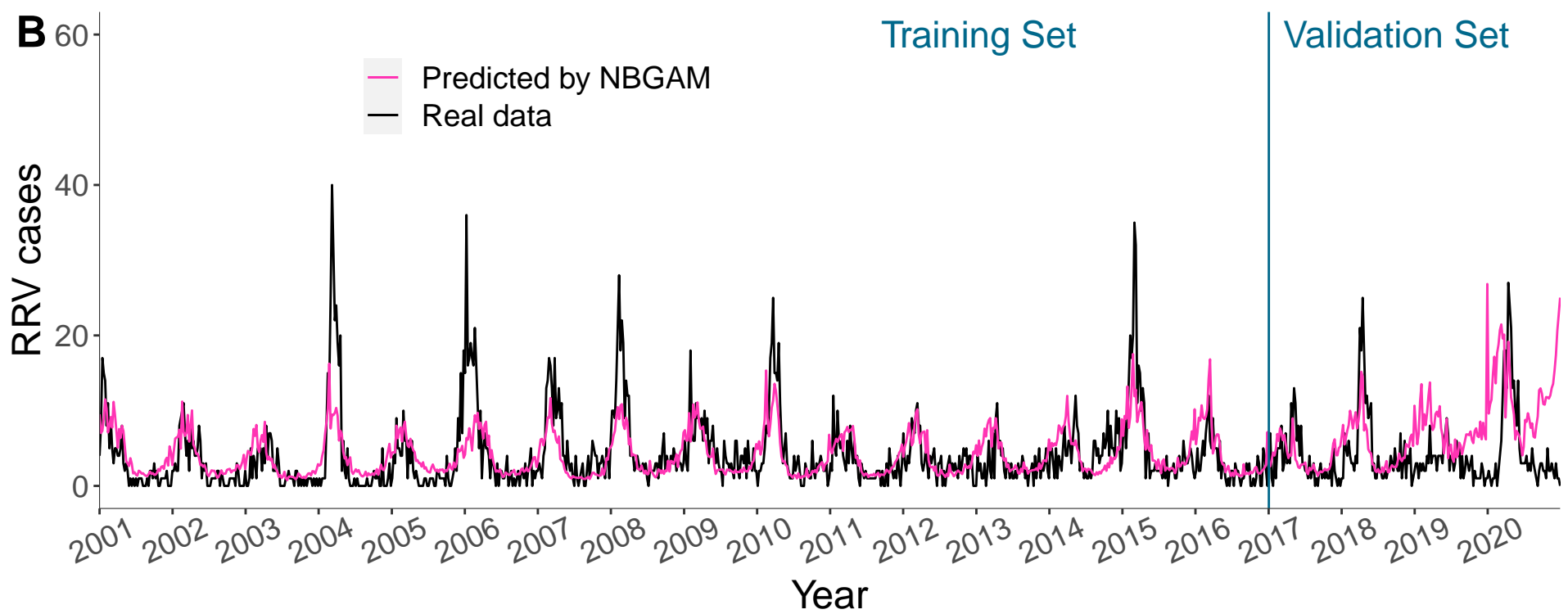

Supplement: Figure S4 [file peerj-10-14213-s007.pdf]

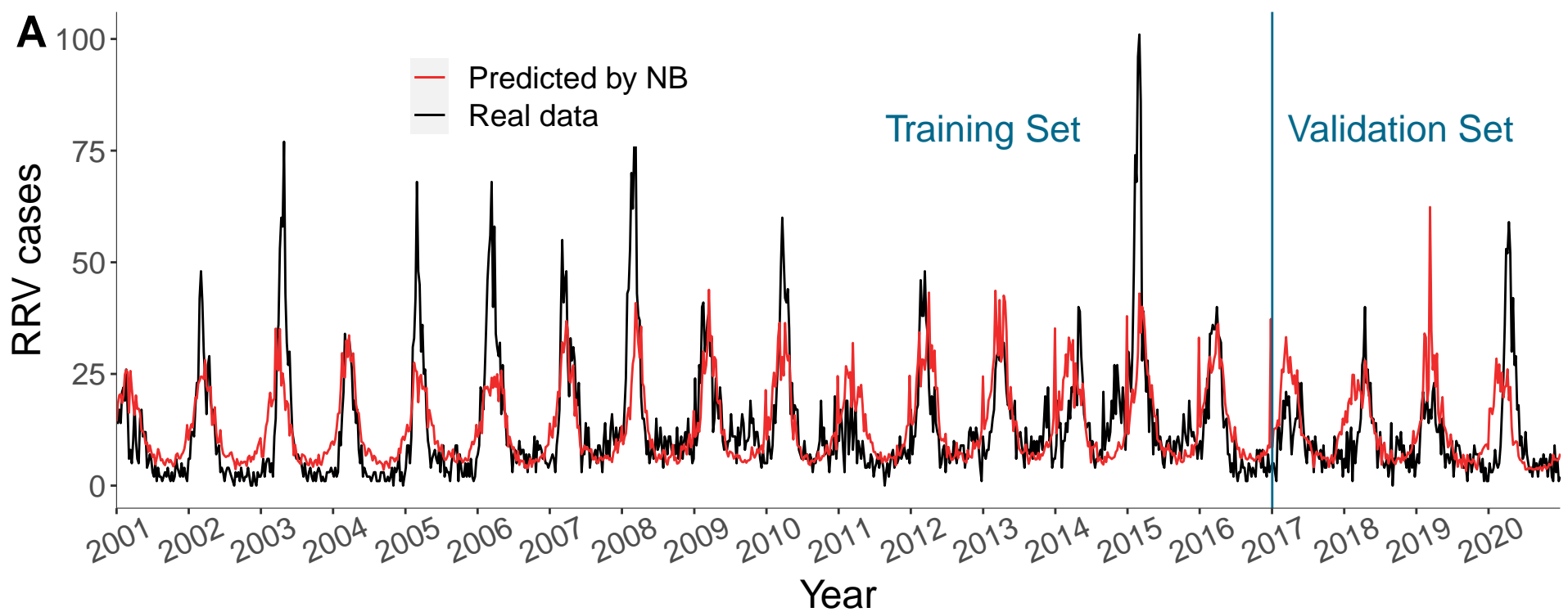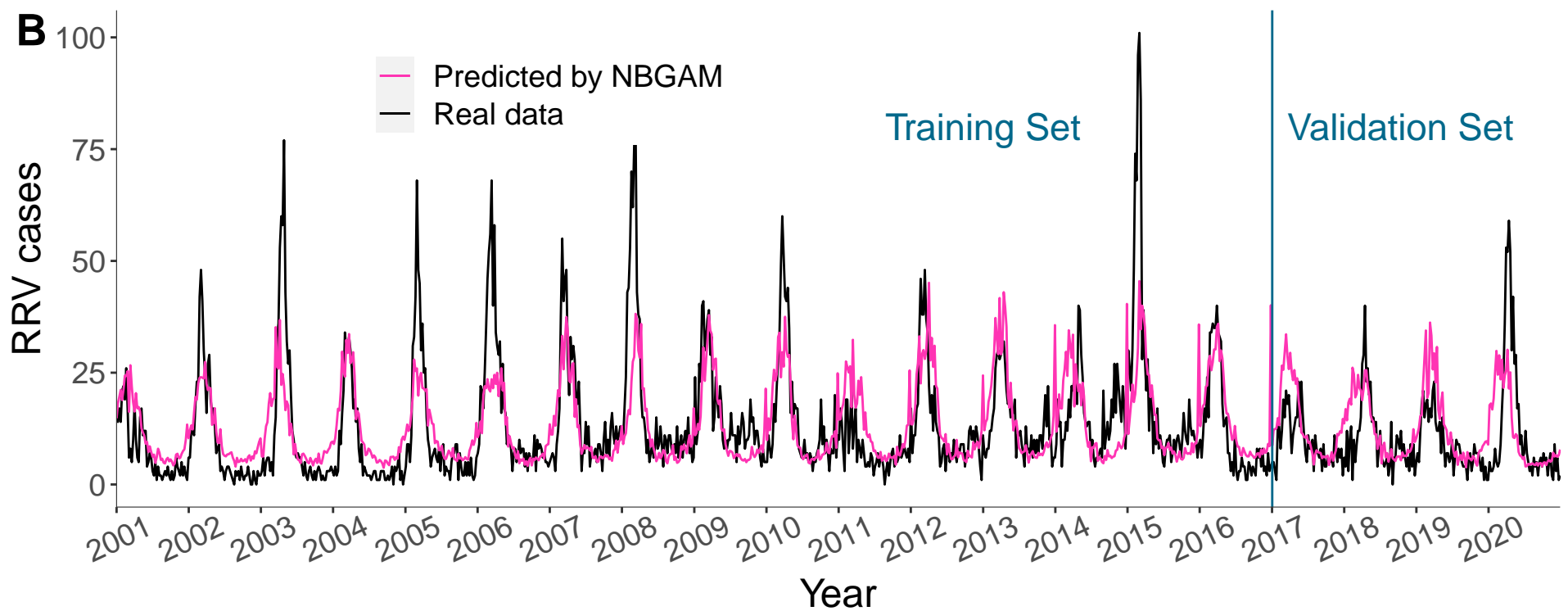

Supplement: Figure S5 [file peerj-10-14213-s008.pdf]

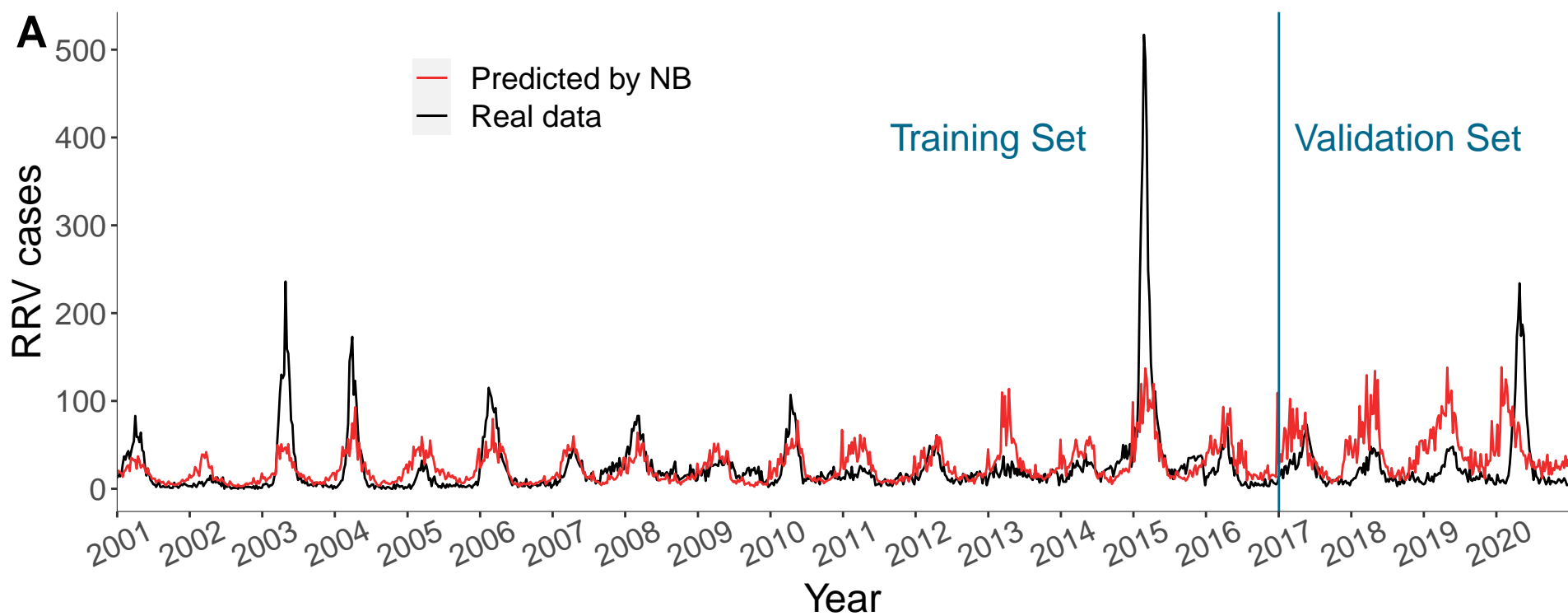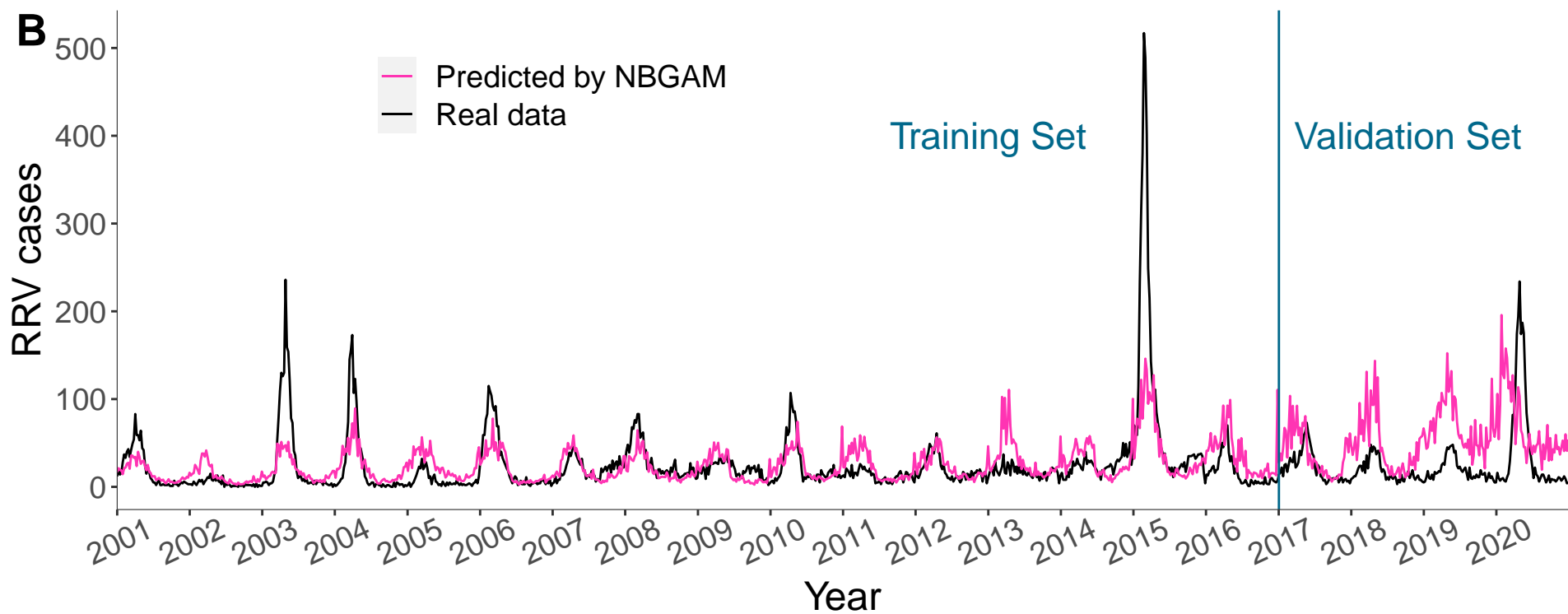

Supplement: Figure S6 [file peerj-10-14213-s009.pdf]

Linear models    ZI models    Non-linear models

All

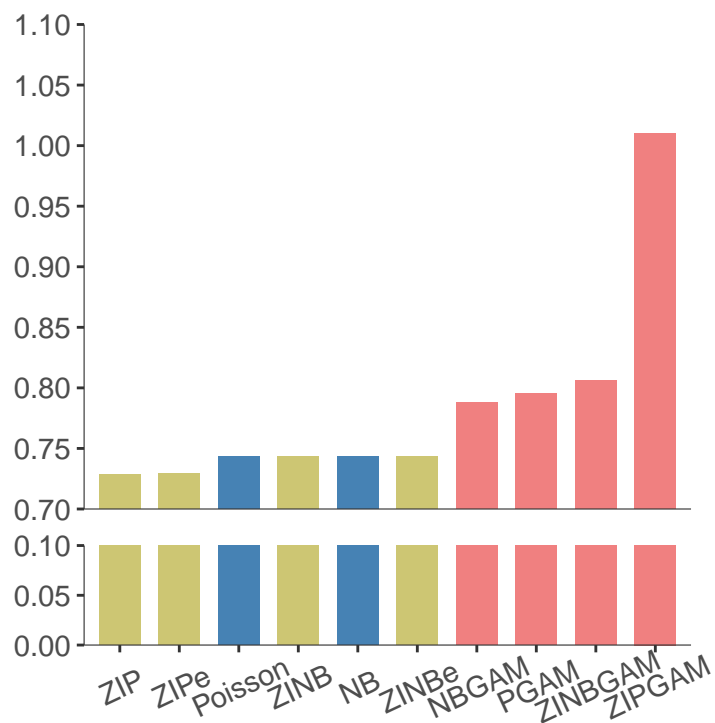

Warm

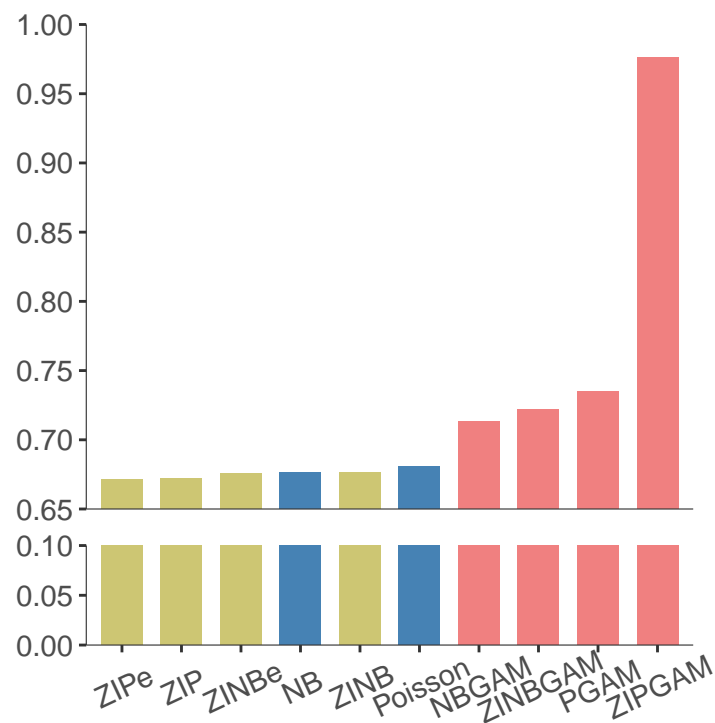

Dry

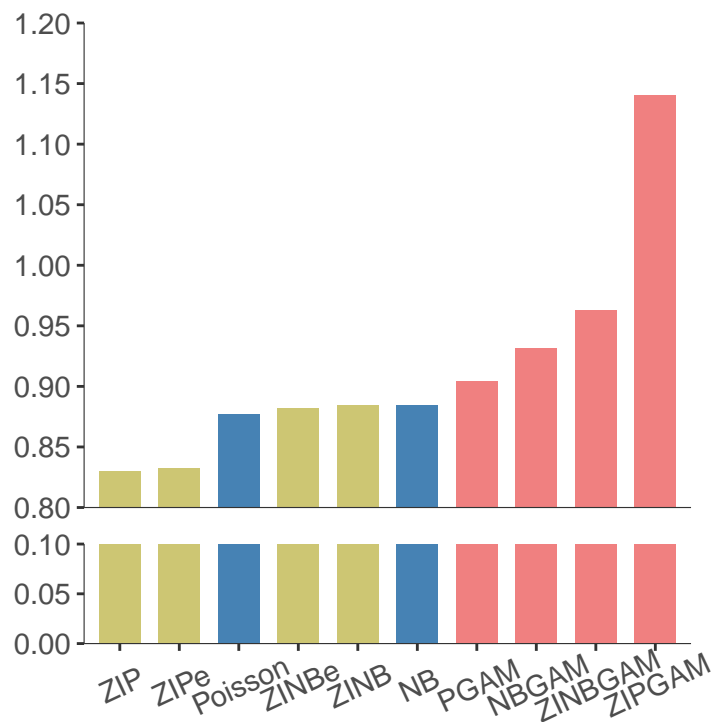

Hot

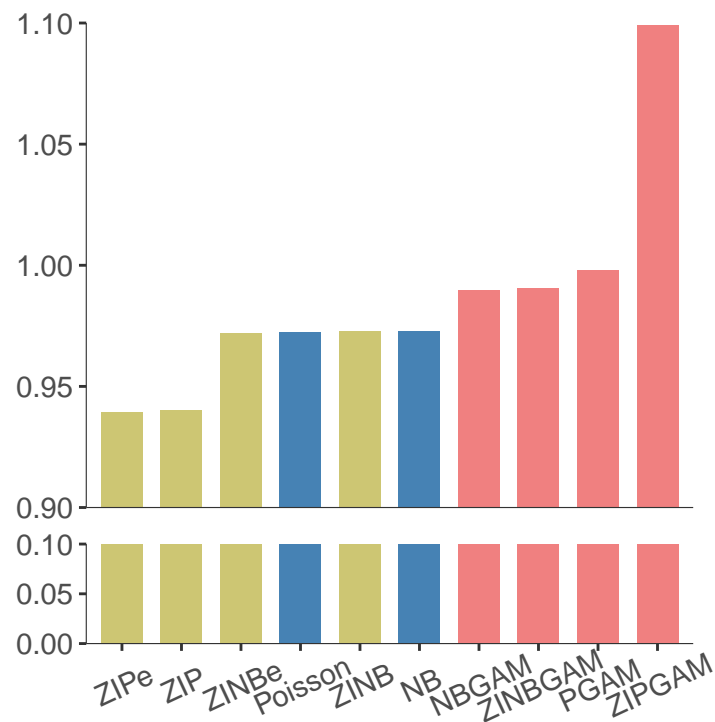

Method

Supplement: Figure S7 [file peerj-10-14213-s010.pdf]
